# Risk of postoperative pulmonary complications in adult surgical patients with metabolic syndrome: a systematic review and meta-analysis protocol

**DOI:** 10.1186/s13643-019-1241-z

**Published:** 2019-12-06

**Authors:** Philip Norris, Bianca Viljoen, Nicholas Ralph, Jeff Gow, Natalie Silvey

**Affiliations:** 1 School of Nursing and Midwifery, Faculty of Health, Engineering and Sciences, University of Southern Queensland, Toowoomba, Australia; 2 School of Commerce, Faculty of Business, Education, Law and Arts, University of Southern Queensland, Toowoomba, Australia; 3 School of Accounting, Economics and Finance, Durban, South Africa; 4 Imperial School of Anaesthesia, London, UK

**Keywords:** Metabolic syndrome, Surgery, Complications, Postoperative pulmonary complications, Systematic review, Meta-analysis, Protocol

## Abstract

**Background:**

Metabolic syndrome (MetS) is defined as an accumulation of risk factors that include chronic hypertension, dyslipidaemia, insulin resistance and obesity and leads to an increased risk for diabetes, cardiovascular disease and stroke. MetS is widespread and estimated to affect up to a quarter of the global population. Patients with MetS who undergo surgery are associated with an increased risk of postoperative complications when compared with patients with a non-MetS profile. An emerging body of literature points to MetS being associated with a greater risk of postoperative pulmonary complications (PPC) in the surgical patient. PPC are associated with increased postoperative morbidity and mortality, Intensive care unit (ICU) admission, length of stay (ICU and hospital), health care costs, resource usage, unplanned re-intubation and prolonged ventilatory time.

**Methods/design:**

We will search for relevant studies in the following electronic bibliographic databases: EMBASE, MEDLINE, Cumulative Index to Nursing and Allied Health Literature (CINAHL) and Scopus as well as scan the reference lists of included studies for potential additional literature. Two authors will independently screen titles and abstracts to identify potentially relevant studies for inclusion based on predefined inclusion and exclusion criteria. The Cochrane Collaboration Review Manager (Review Manager 5) statistical software will be used to conduct this systematic review and meta-analysis and generate forest plots to demonstrate comparison of findings across studies included for meta-analysis. Subgroup and sensitivity analysis will be performed to assess the heterogeneity of included studies. A descriptive synthesis of the statistical data will be provided to summarise the results and findings of the systematic review and meta-analysis.

**Discussion:**

This review will be the first to report and summarise the risk for and incidence of PPC in adult patients with MetS undergoing surgery across a range of surgical specialities. The results have the potential to inform the development of evidenced-based interventions to improve the management of PPC in the surgical patient with MetS. Findings from this systematic review and meta-analysis will inform a subsequent Delphi study on priorities and responses to PPC in patients with MetS. We will also disseminate our results through publication in scientific peer-reviewed journals, conference presentations and promotion throughout our network of surgical safety champions in clinical settings.

**Systematic review registration:**

PROSPERO CRD42019120279.

## Background

Metabolic syndrome (MetS) is a global health problem which leads to an increased risk of cardiovascular disease, stroke, non-insulin dependent diabetes mellitus and premature death [1]. Individuals with MetS typically present with symptoms that include elevated blood pressure, insulin resistance, decreased high-density lipoproteins, elevated triglycerides and obesity, particularly abdominal obesity [2, 3]. Typically, an individual is diagnosed with MetS when at least three out of five of these components are present [4, 5]. However, these components vary slightly according to the definition of MetS used. While the various definitions share the same inherent risk factors, diagnostic criteria diverge slightly across each definition. Historically, two commonly used definitions are from the International Diabetes Federation (IDF), and the National Cholesterol Education Program Adult Treatment Panel III (NCEP:ATPIII) [6, 7]. To overcome the difficulties associated with multiple definitions of MetS, leading health organisations produced a Joint Interim Statement (JIS) to harmonise diagnostic criteria and components of MetS into a unified, widely accepted, and broadly adopted definition as outline in Table 1 [6].

**Table 1:** Diagnostic criteria for the metabolic syndrome

Measure	Categorical cut–off points
Elevated waist circumference	Population- and country-specific definitions
Elevated triglycerides (drug treatment for elevated triglycerides is an alternate indicator)	≥ 150 mg/dL (1.7 mmol/L)
Reduced HDL-C (drug treatment for reduced HDL-C is an alternate indicator)	< 40 mg/dL (1.0 mmol/L) in males; < 50 mg/dL (1.3 mmol/L) in females
Elevated blood pressure (antihypertensive drug treatment in a patient with a history of hypertension is an alternative indicator	Systolic ≥ 130 and/or diastolic ≥ 85 mmHg
Elevated fasting glucose (drug treatment of elevated glucose in an alternate indictor)	≥ 100 mg/dL

Unifying and using a single diagnostic criteria for MetS is important as previous global estimates of prevalence range between 10% and 84% depending on region, age, ethnicity, gender and race [1]. However, MetS is most commonly estimated to be around 20 to 30% in adult populations within developed economies [8, 9]. While an accurate prevalence of MetS in surgical patients is not obtainable from the literature, the prevalence of MetS in patients undergoing cardiac surgery alone is estimated at approximately 46% [10, 11]. This estimate is almost double that reported in the general population [12, 13]. A diagnosis of MetS predicts the development of chronic disease and deterioration of health. MetS increases the risk for all-cause mortality 1.5 fold, stroke twofold, cardiovascular disease threefold, and type two diabetes mellitus fivefold [1, 14–16]. MetS also predicts the development of a range of chronic diseases including non-alcoholic steatohepatitis, neurological disorders and malignant neoplasms [17]. Having MetS is further associated with increased complications in patients undergoing surgery compared to patients with a non-MetS profile. Frequently reported perioperative adverse events indicate that patients with MetS are associated with increased rates of postoperative morbidity compared to patients without it specifically, infectious, cardiovascular and renal postoperative adverse events [10, 18, 19].

One particular under-researched area of burden predicted by MetS relates to postoperative pulmonary complications (PPC) after surgery. PPC occur commonly and increase patient mortality and morbidity as well as being associated with increased health-care costs. One in five patients who develop a PPC will die within 30 days of major surgery compared to 0.2–3% without [20] and an observational study has shown that there are long-term significant differences in mortality rates at 1 and 5 years for patients who have had a PPC [20]. Length of hospital stay is also increased with resultant increased morbidity and this also increases healthcare costs significantly [21]. Increasingly, there are reports of a heightened risk of PPC among surgical patients’ with MetS. For example, in a study of 158,405 patients undergoing bariatric surgery, the complications of atelectasis, pleural effusions, pneumonia, ARDS and respiratory failure were associated with a significantly higher percentage of occurrences in patients with MetS compared to patients without a MetS diagnosis [22]. For patients undergoing hepatic surgery, having MetS increases the risk of unplanned re-intubation and prolonged ventilatory time twofold [23]. In a study reporting on outcomes for patients undergoing cardiac surgery, rates of PPC and length of ventilatory time were significantly higher in patients with a MetS profile [24]. In another retrospective cohort study of 310,280 patients undergoing non-cardiac surgery, the risk for PPC was approximately 1.5 to threefold higher in patients when accompanied by a MetS diagnosis [25]. MetS was found to be an independent positive predictor of PPC in patients undergoing abdominal surgery [26]. Increased rates of PPC were also reported in patients diagnosed with MetS who underwent lumbar spinal fusion and shoulder arthroplasty [27, 28]. An emerging body of literature appears to associate MetS with a greater risk of PPC in the surgical patient. However, no systematic review has been performed to date meaning the risk of PPC in MetS patients is not well understood. Additionally, there are few studies of interventions specifically targeting the needs of the surgical patient with MetS. Our proposed systematic review and meta-analysis will therefore be the first of its kind to synthesise and critically appraise the current evidence base. We will quantify the risk of PPC in patients with metabolic syndrome versus those without metabolic syndrome in surgical populations.

## Objectives

The objective of this review is to evaluate the effect of metabolic syndrome on the occurrence of PPC in adult surgical patients versus those without metabolic syndrome.

## Methods

### Protocol

This systematic review will follow the Preferred Reporting Items for Systematic Review and Meta-Analysis (PRISMA) recommendations [29]. The systematic review protocol has been registered in the International Prospective Register of Systematic Reviews (PROSPERO CRD42019120279). A PRISMA-P file is attached (see Additional file 1) [30].

### Eligibility criteria

#### Criteria for considering studies

##### Study inclusion criteria

This review will include peer-reviewed literature that is published in electronic bibliographic databases. Included studies must examine the relationship between adult surgical patients 18 years of age and older diagnosed with MetS and the risk for and incidence of PPC in this population. Studies will be restricted by design with only retrospective and prospective observational studies, cohort studies and case-control studies being eligible for inclusion to obtain a comprehensive overview of the existing evidence base.

#### Population

Surgical patients diagnosed with MetS versus surgical patients without MetS.

#### Outcomes

The primary outcome will be the incidence of PPC in adult surgical patients diagnosed with MetS at key surgical/clinical time intervals. We will report outcomes of interest as odds ratio, relative risk or incidence rate ratios. We will only record outcomes reported within the first 30 days after surgery.

PPC will be defined using the European Perioperative Clinical Outcomes (EPCO) definitions [31]. These include respiratory infection, respiratory failure, pleural effusion, atelectasis, pneumothorax, bronchospasm, aspiration pneumonitis, pneumonia, acute respiratory distress syndrome (ARDS), tracheobronchitis, pulmonary oedema, exacerbation of pre-existing lung disease and pulmonary embolism.

We will also record secondary outcomes of interest as follows:

- Unplanned re-intubation within 30 days
- Prolonged ventilatory time > 72 h

### Search strategy

We will conduct a comprehensive literature search of the following electronic databases: MEDLINE (Medical Literature Analysis and Retrieval Online), CINAHL (Cumulative Index to Nursing and Allied Health Literature), EMBASE (ExerptaMedica Database) and Scopus to identify studies pertinent to the review. A date restriction from January 1, 1998 until present, will be implemented to reflect the first formal definition for metabolic syndrome which was developed in 1998 by the World Health Organization and informed every subsequent definition of MetS since [6].

The types of studies that will be analysed and included are retrospective or prospective observational studies, cross-sectional studies, cohort studies and case control studies. The types of studies that will be excluded are letters, abstracts, conference papers and editorials. The initial search terms reflect how metabolic syndrome is commonly referred to in the literature. These keywords will include the Medical Subject Heading for “metabolic syndrome” and a range of synonyms used to represent surgery and post-operative pulmonary complications. Search terms will be limited to peer-reviewed full text-articles, in English language with no geographical restriction (see Appendix 1).

To ensure that all relevant studies are included, a manual search of citations and references of eligible studies will also be conducted. Resulting references will be exported separately and provided to two reviewers (PN and BV) for independent review. Where necessary, study authors will be contacted for missing information. To ensure impartiality the inclusion and exclusion criteria will be engaged continuously (see Appendix 2).

### Selection of studies

Following the search, studies selected for inclusion will be collated into a citation management program (Endnote X8). Duplicates will be removed and stored separately (Endnote X8). Two reviewers (PN and BV) will independently screen titles and abstracts for assessment against the inclusion criteria and exclude all studies that do not meet these criteria. Any disagreement will be resolved through discussion and consensus. After the initial screening for studies, two reviewers (PN and BV) will retrieve the full text of selected studies and assess against the inclusion criteria. Full-text studies that meet the inclusion criteria will be imported into Endnote X8. Full text studies that do not meet the inclusion criteria will be excluded and reasons for exclusion will be provided in an appendix in the final systematic review report. Included studies will undergo a process of critical appraisal. Any disagreements that arise between the reviewers will be resolved through discussion, or with a third reviewer (NR). The results of the search will be reported in the review and presented in a PRISMA flow diagram, detailing the steps taken in the full systematic review. The authors will be contacted for missing data where required.

### Data extraction and management

Two reviewers (PN and BV) will independently extract data from the included studies into Cochrane Collaboration Review Manager (Review Manager 5) to ensure consistency while reducing bias and improving validity and reliability [32]. Any inconsistences between reviewers will be resolved through consensus and a third reviewer will be consulted if agreement cannot be reached. Study authors will be contacted to obtain additional or missing data.

Extracted data will include the following:

1. Study details: title, journal, year, city and country where the research was undertaken.
2. Participant demographics: sample size, group size (e.g. metabolic syndrome group versus non-metabolic syndrome group), reported complications, metabolic syndrome diagnostic criteria applied, type of surgery, population demographics.
3. Methods: study aim, data collection method, study recruitment, design and sampling methods, study eligibility as dictated by the inclusion criteria
4. Outcome measures: estimates of cumulative incidence and incidence rate of PPC in adult surgical patients diagnosed with MetS.
5. Limitations: study biases as identified by a risk of bias tool for observational studies and limitations as identified by the study authors.

### Risk of bias

The methodological quality and bias of the included studies will be assessed using the Newcastle-Ottawa Quality Assessment Scale (NOS) [33]. The NOS will evaluate non-randomised studies included in systematic reviews and meta-analyses across three quality parameters: study selection, comparability of the population and a determination of whether the exposure or outcome includes risk of bias [32]. NOS assesses the quality of each study and provides a maximum score of nine points. Studies identified as having a NOS greater than or equal to seven are considered high quality. Studies between five and six points are considered as being of fair or moderate quality. Studies that are assessed as having a NOS score of less than five points represent a high risk of bias [32]. Two investigators (PN and BV) will independently assess the risk of bias for included studies using NOS quality criteria.

### Data collection and analysis

We will conduct data analysis using the Cochrane Collaboration Review Manager (Review Manager 5) statistical software to generate forest plots to demonstrate comparison of findings across studies included for meta-analysis. Meta-analysis will be performed if two or more comparable studies are identified for each outcome of interest. We anticipate clinical and methodological diversity as population and study characteristics will vary across trials. The statistical heterogeneity of studies included for meta-analysis will be assessed by calculating the *P* value from the chi-squared test for homogeneity and the *I*^2^ statistic. A *P* value of < 0.10 will be used to determine statistical significance [28]. In the presence of substantial heterogeneity (*I*^2^ is ≥ 50%), we will pool study-specific estimates using a random effects model and according to whether variables are dichotomous or continuous [34]. For dichotomous variables, individual study data will be pooled and examined using the Mantel-Haenszel to examine the overall association between the exposure and outcome [35]. For continuous variables, we will group measures using the inverse variance approach to pool the standardised mean difference where studies have employed different measures to calculate the outcome of interest or weighted mean difference if studies report efficacy in terms of a continuous outcome measurement [36].

If heterogeneity is low (*I*^2^ is ≤ *5*0%), pooled estimates will be calculated using a fixed-effects model [36]. Measures of relative effect will be expressed as odds ratio (OR) and 95% confidence intervals (CIs) for dichotomous outcomes while standard mean differences and 95% CIs will be calculated for continuous outcomes. If statistical heterogeneity is present, subgroup analysis (e.g. population according to the MetS definition categories, surgery type, type of PPC, age and gender) will be undertaken and meta-regression performed to explore effect measure modification where the modifiers are study level covariates. Sensitivity analysis will be performed to identify study level categorical variables that characterise the occurrence of the outcome of interest. We will also assess the impact of outlier studies on the pooled estimates of reported outcome measures among the population of interest by performing outlier analysis.

If there are ten or more studies included in the meta-analysis, the reviewers will assess for potential publication bias using funnel plots and Egger’s test [37]. Any discrepancies of quality assessment between the two reviewers (PN and BV) will be resolved through discussion and consensus or by a third reviewer (NR).

## Discussion

To our knowledge, this systematic review and meta-analysis will be the first to evaluate the risk of PPC following surgery in patients with and without MetS. If MetS increases the probability of complications following surgery, patients should be made aware of these risks while clinicians should develop interventions to reduce or eliminate the likelihood of PPC occurring. Following our review, we will use numerous strategies to disseminate our findings. Examples include conference presentations, media releases, meetings with healthcare leaders and expansion of our current research program in metabolic syndrome [16] to include PPC intervention development. We will also use findings from our review to inform a subsequent Delphi study as described in [38] to identify expert consensus on priorities for improving outcomes among surgical patients with metabolic syndrome.

### Supplementary information

**Additional file 1.** PRISMA-P Checklist.

## Data Availability

Not applicable as no datasets have been compiled as of yet.

## Appendix 1

**Table 2:** Example search strategies

Search Date	MEDLINE search strategy
January 06, 2019	((“surgical procedures, operative”[MeSH Major Topic] OR “surgery”[Title/Abstract]) AND (“metabolic syndrome”[MeSH Major Topic] OR “metabolic syndrome”[Title/Abstract])) AND (((“lung”[MeSH Major Topic] OR “respiratory therapy”[MeSH Major Topic]) OR “lung”[Title/Abstract]) OR “pulmonary”[Title/Abstract])AND (“1998/01/01”[PDAT]: “2019/01/18”[PDAT]) AND (“1998/01/01”[PDAT]: “2019/01/18”[PDAT])
Search Date	CINAHL search strategy
January 06, 2019	(((TI Metabolic syndrome) OR (AB metabolic syndrome)) AND (((TI surgical procedures) OR (AB surgical procedures) OR ((TI operative) OR (AB operative)) OR ((TI surgery) OR (AB surgery) AND (((TI Metabolic syndrome) OR (AB metabolic syndrome)) AND (((TI respiratory therapy) OR (AB respiratory therapy)) OR ((TI lungs) OR (AB lungs)) OR ((pulmonary) OR (AB pulmonary))) with limiters of a published date from 1998/01/01
Search Date	ScienceDirect search strategy
January 06, 2019	title-abs-key (metabolic syndrome) AND title-abs-key (surgical procedures*) OR title-abs-key (operative*) OR title-abs-key (surgery*) AND title-abs-key (respiratory therapy*) OR title-abs-key (lung) OR title-abs-key (pulmonary) AND LIMIT-TO (yearnav, “2019, 2018, 2017, 2016, 2015, 2014, 2013, 2012, 2011, 2010, 2009, 2008, 2007, 2006, 2005, 2004, 2003, 2002, 2001, 2000, 1999, 1998”) AND LIMIT-TO (contenttype, “JL, BS”, “Journal”).
Search Date	Embase Search strategy
January 06, 2019	‘metabolic syndrome’:ab,ti AND surgical procedures: ab,ti OR operative: ab,ti OR surgery ab,ti AND pulmonary:ab,ti OR respiratory therapy:ab,ti OR lungs ab.ti[1998–2019]/py AND [english]/lim AND [humans]/lim

## Appendix 2

**Table 3:** Example inclusion and exclusion criteria

Example inclusion criteria	Example exclusion criteria
Study population • Adult patients (18 years or older) • Adult patients with a diagnosis of metabolic syndrome as per author definition • Adult patients undergoing moderate or highly invasive surgery of all types Study surgical procedure • Adult patients undergoing moderate or highly invasive surgery of all types Study design • Observational studies (e.g. cohort studies, case-control studies) • Published peer-reviewed articles in the English language	Study population • Surgical patients < 18 years of age • Animals • Pregnant women Study surgical procedure • Minor procedures • Caesarean section • Trauma surgery • Day surgical procedures • Cataract procedures • Plastic surgery • Cosmetic surgery • Cardiac catheterisation • Cystoscopy procedures • Endoscopy procedures • Colonoscopy procedures • Lesion removal Study design • Interventional studies • Conference procedures • Narrative reviews • Editorials • Lectures and presentations • Articles in a language other than English

## Abbreviations

ARDS: Acute respiratory distress syndrome
CABG: Coronary artery bypass graft
CINAHL: Cumulative Index to Nursing and Allied Health Literature
CIs: Confidence intervals
EPCO: European Perioperative Clinical Outcomes
HDL: High-density lipoprotein
ICU: Intensive care unit
IDF: International Diabetes Federation
JIS: Joint Interim Statement
MetS: Metabolic syndrome
NCEP:ATPIII: National Cholesterol Education Program Panel III
NOS: Newcastle Ottawa Scale
OR: Odds ratio
PPC: Postoperative pulmonary complications
PRISMA-P: Preferred Reporting Items for Systematic Review and Meta-Analysis Protocols
